# Clinically feasible method for assessing leukocyte rheology in whole blood

**DOI:** 10.1007/s00380-019-01486-y

**Published:** 2019-08-23

**Authors:** Riha Shimizu, Hirotsugu Fukuda, Yuji Kikuchi, Hirokazu Yanaka, Nobuhiro Hata, Masashi Yamazaki, Yuki Nakatani, Yuma Tamura, Seiko Yamakoshi, Atsuhiko Kawabe, Yasuto Horie, Hiroyuki Sugimura, Yasushi Matsushita, Takaaki Nakamoto, Takanori Yasu

**Affiliations:** 1 Department of Cardiac and Vascular Surgery, Dokkyo Medical University Nikko Medical Center, Nikko, Tochigi Japan; 2 Department of Cardiac and Vascular Surgery, Dokkyo Medical University, Mibu, Tochigi, Japan; 3 Kikuchi Microtechnology Institute, Ryugasaki, Ibaraki Japan; 4 Department of Clinical Laboratory, Dokkyo Medical University Nikko Medical Center, Nikko, Tochigi Japan; 5 National Institute of Advanced Industrial Science and Technology, Tsukuba, Ibaraki Japan; 6 Department of Diabetes and Endocrinology, Dokkyo Medical University Nikko Medical Center, Nikko, Tochigi Japan; 7 Department of Rehabilitation, Dokkyo Medical University Nikko Medical Center, Nikko, Tochigi Japan; 8 Department of Cardiovascular Medicine and Nephrology, Dokkyo Medical University Nikko Medical Center, 632 Takatoku, Nikko, 321-2593 Tochigi Japan; 9 Department of Cardiology, Dokkyo Medical University Nikko Medical Center, Nikko, Tochigi Japan

**Keywords:** Acute coronary syndrome, Diabetes mellitus, Leukocyte, Microcirculation, Rheology

## Abstract

This study reports a novel method for assessment of leukocyte rheological activation with a new designed microchannel array chip to mimic the human microvascular network for microchannel array flow analysis (MCFAN). Study subjects were 79 healthy volunteers and 42 patients with type 2 diabetes mellitus (DM) and 36 patients with acute coronary syndrome (ACS). Using the anticoagulants heparin and ethylene-diamine-tetraacetic acid (EDTA)-2Na which inhibits platelets and leukocytes by chelating Ca^2+^, we were able to quantify leukocyte rheological activation by the subtraction of passage time of blood treated with both heparin and EDTA-2Na from that of blood treated with heparin only. We confirmed that passage times of whole blood with heparin + EDTA-2Na were always shorter than those of whole blood with only heparin in healthy subjects and patients with DM or ACS under suction pressures of − 30 cmH_2_O. There was a significant correlation between delta whole blood passage time {(heparin tube) − (EDTA-2Na + heparin)} and serum levels of myeloperoxidase and adhesive leukocyte number, respectively, even in blood from patients with DM or ACS, who suffered from inflammation. In conclusion we have developed a clinically feasible method for assessing leukocyte rheological activation in whole blood in ex vivo.

**Electronic supplementary material:**

The online version of this article (10.1007/s00380-019-01486-y) contains supplementary material, which is available to authorized users.

## Introduction

Abnormal activation states of leukocytes and leukocyte–platelet interactions play key roles in organ injury induced by atherosclerotic disease [1, 2], diabetes mellitus, and other inflammatory conditions [3–5]. Leukocyte rheology is critical for modulating microvascular haemodynamics through transformation from resting to active states under conditions of inflammation or low shear stress [6–9], such as high-fat diets [4], triggered by free fatty acids [5–7] and ischemia/reperfusion. Decreased leukocyte deformability and increased leukocyte adherence to the post-capillary venular endothelium leads to microvascular dysfunction, in part, through increased blood viscosity [5–7]. Because of its larger volume and higher cellular viscosity, each leukocyte is equivalent to approximately 700 erythrocytes in its tendency to block 5 μm capillary channels [8]. The rheology of leukocytes has significant implications on their functional behaviour, including flow-through capillaries and interactions with endothelial cells [5–10].

Cohort studies have shown blood viscosity to be a strong predictor of cardiovascular events [11], particularly in diabetic patients [12, 13]. However, clinically feasible methods for determining leukocyte activation and the hemorheological character of blood, a non-Newtonian fluid, are limited. A microchannel flow analyser (MCFAN) with a conventional siliconized chip (BK 7-7-4.5D) is a generally accepted ex vivo capillary model for the evaluation of whole blood rheology [5–7, 14–16]. However, the BK 7-7-4.5 model differs from in vivo microvessels in its abrupt narrowing and shorter capillary length. In conventional microarray chips with abrupt narrowing, we have observed platelet aggregation and platelet–leukocyte adhesion at the post-capillary venules and plugging of some terminal capillaries, possibly due to activation of glycoprotein IIb/IIIa on platelets caused by the abrupt increase in shear stress [17]. This artefactual activation of platelets downstream of capillaries may prolong the whole blood passage time somewhat.

Therefore, we designed and manufactured a new microchannel array chip, DKAMCM1-60-7-4.5D, to mimic the human microvessel network. Stepwise increases in shear stress may prevent artefactual activation of platelets. The silicon dioxide film on the silicon wafers provides phase contrast that permits clear microscopic observation of blood cells, especially transparent leukocytes and platelets, from passing through the microchannel array without dying [18].

Although the number of adhesive leukocytes per unit area has been reported as an index of leukocyte activation [5–7], its use is not clinically feasible because the measurement is time-consuming. EDTA-2Na scavenges metal ions (Ca^2+^ and Mg^2+^) essential to the activation of platelets and some leukocytes [8, 19]. Therefore, we postulate that the change in passage time between heparinized blood and EDTA-2Na + heparinized blood may reflect leukocyte rheology in whole blood in vivo. To explore clinically feasible indicators of leukocyte activity, as well as blood rheology using MCFAN, with the newly developed siliconized microchannel chip, we examined the correlation between the number of adhesive leukocytes per field of heparinized blood, plasma levels of myeloperoxidase (MPO), and the change of whole blood passage time {(heparinized blood) − (EDTA-2Na + heparinized blood)} under a constant vacuum of 30 cmH_2_O and 60 cmH_2_O after calibration, according to the duration of saline passage through the channels.

## Materials and methods

### Study participants

The clinical characteristics of the study participants including 79 healthy volunteers, 42 patients with type 2 diabetes mellitus (DM) and 36 patients with acute coronary syndrome (ACS) are shown in Table 1. Seventy-nine healthy, Japanese volunteers did not have any history of DM, hypertension, dyslipidaemia, collagen disease, cardiopulmonary disease, liver and kidney disease, or malignancy; were not currently pregnant or on any medication; and their results of routine physical examinations and standard laboratory tests were normal. Emergent coronary arterial intervention was performed in all the 36 patients with acute coronary syndrome within 10–14 days.

**Table 1:** Clinical characteristics of study subjects

	Healthy subjects (*n* = 79)	Diabetes mellitus (*n* = 42)	Acute coronary syndrome (*n* = 36)
Age (years)	36.6 ± 10.9	64.4 ± 8.5	65.5 ± 10.1
Sex (male)	39	30	32
Height (cm)	165.1 ± 8.6	161.5 ± 9.4	161.9 ± 8.0
Weight (kg)	61.3 ± 12.4	70.3 ± 11.2	64.4 ± 10.2
BMI (kg/m^2^)	22.4 ± 3.5	27.1 ± 4.6	24.5 ± 3.1
Current smoker (%)	1 (1.3%)	1 (2.3%)	14 (38.9%)
White blood cells (/μL)	6462 ± 1356	6227 ± 1601	6628 ± 1908
Haemoglobin (g/dL)	14.0 ± 1.7	14.2 ± 1.4	13.3 ± 1.5
Haematocrit (%)	43.1 ± 4.3	41.7 ± 4.2	39.1 ± 4.1
Platelets (10^3^/μL)	26.1 ± 5.0	21.1 ± 5.2	25.1 ± 7.7
LDL cholesterol (mg/dL)	102.8 ± 33.1	86.9 ± 32.1	84.2 ± 24.6
HDL cholesterol (mg/dL)	60.9 ± 18.3	49.5 ± 11.1	39.1 ± 13.0
Triglycerides (mg/dL)	94.5 ± 83.7	150.8 ± 88.9	138.9 ± 80.2
Plasma glucose (mg/dL)	87.7 ± 13.2	157.0 ± 52.0	133.7 ± 58.7
eGF min R (mL/ /1.73)	91.6 ± 18.7	69.7 ± 18.4	62.3 ± 17.7
HbA1c (%)	5.2 ± 0.3	7.9 ± 1.0	6.7 ± 1.7
BNP (pg/mL)		32.5 ± 39.0 (median 11.6)	71.0 ± 50.8 (median 55.8)

Data are expressed as the mean ± SD

*BMI* body mass index, *LDL* low-density lipoprotein, *HDL* high-density lipoprotein, *eGFR* estimated glomerular filtration rate, *HbA1c* haemoglobin A1c, *SD* standard deviation, *BNP* brain natriuretic peptide

### Blood kinetics in an ex vivo microchannel microvascular model

We used a plot-type microchannel flow analyser system (BWA-MCFAN, Kikuchi Microtechnology Co., Ltd. Ibaraki, Japan) equipped with a new microchannel array chip (DKAMCM1-60-7-4.5D, National Institute of Advanced Industrial Science and Technology, Tsukuba, Ibaraki, Japan) designed as an ex vivo microvascular model to assess whole blood rheology and leukocyte activity. The detailed procedures and apparatus of BWA-MCFAN have been described elsewhere [14, 15]. In short, microgrooves formed on the surface of a silicon chip were converted to leak-proof microchannels by covering them tightly with an optical flat glass plate in a holder. The contact between the two surfaces could be made watertight by mechanical pressing alone because of their optical flatness. The microgrooves in the silicon microchannel chip were prefilled with saline.

Within 20 min of collecting blood into the two tubes, 0.1 mL of blood was drawn through the chip under a constant vacuum of − 30 cmH_2_O (2.94 kPa, physiological pressure difference between arterioles and venules) or − 60 cmH_2_O (5.88 kPa, pathological pressure difference between arterioles and venules). The time required for saline to pass through the microchannels was determined before each blood measurement for calibration. Microscopic motion images of blood passing through microchannels were monitored and stored via computer. When 0.08–0.10 mL of blood had exited the microchannel array, five fields were recorded, five still images were randomly selected for offline analysis, and the numbers of adhesive or clumped leukocytes on the microchannel platform in these images were counted [5–7] by middle-powered field (× 700). Adhesive leukocytes were defined as static leukocytes with a clear surface border on still images [5–7]. The whole blood passage time was normalized as follows: Normalized whole blood passage time = passage time of 100 μL of blood (sec) * × 12 s/passage time of 100 μL of saline (s). Plasma levels of MPO were determined using an enzyme-linked immunosorbent assay (ELISA) kit (Bio Check, Inc., Foster City, CA, USA) [6].

### Study protocol

The study protocol was approved by the Ethical Committee of Dokkyo Medical University Nikko Medical Center. All procedures performed in this study were conducted with informed consent of the patients and complied with the national ethical guidelines for medical and health research involving human subjects and with the 1964 Helsinki Declaration and its later amendments or comparable ethical standards.

All participants arrived in the laboratory at 2:00 pm. The participants had abstained from alcohol, caffeine, and smoking for at least 12 h prior to the study. All participants drank 200 mL of water and remained seated at rest for 5 min before blood sampling. Blood (10 mL) was sampled from the antecubital vein, and 5 mL was collected into each of two tubes: a 5% vol heparin tube (Venoject II, Terumo, Tokyo, Japan) and an EDTA-2Na + 5% vol heparin tube (Venoject II, Terumo, Tokyo, Japan). FMLP (Sigma-Aldrich, Tokyo, Japan) was used to induce leukocyte activation experimentally [19, 20]. FMLP is a chemotactic peptide derived from bacterial protein degradation and mitochondrial proteins upon tissue damage [21, 22] and is present in low concentrations in the bloodstream during inflammation [21, 22]. FMLP was dissolved in dimethyl sulfoxide (Sigma-Aldrich). FMLP (10^–9^ or 10^–10^ M) was added into whole blood from healthy subjects (*n* = 10) in the heparinized tube and EDTA-2Na + heparin tube. After 5 min incubation with FMLP, the whole blood passage time was measured by BWA-MCFAN.

### Statistical analysis

Data were statistically analysed using JMP 14.0 J software (SAS Institute, Cary, NC, USA). Continuous variables are described as the mean ± standard deviation (SD). Differences were analysed with Student's *t* test or 2-way analysis of variance. Correlations were assessed using Fisher’s coefficient (*r*). A *p *value < 0.05 was considered statistically significant.

## Results

**Fig. 1:**
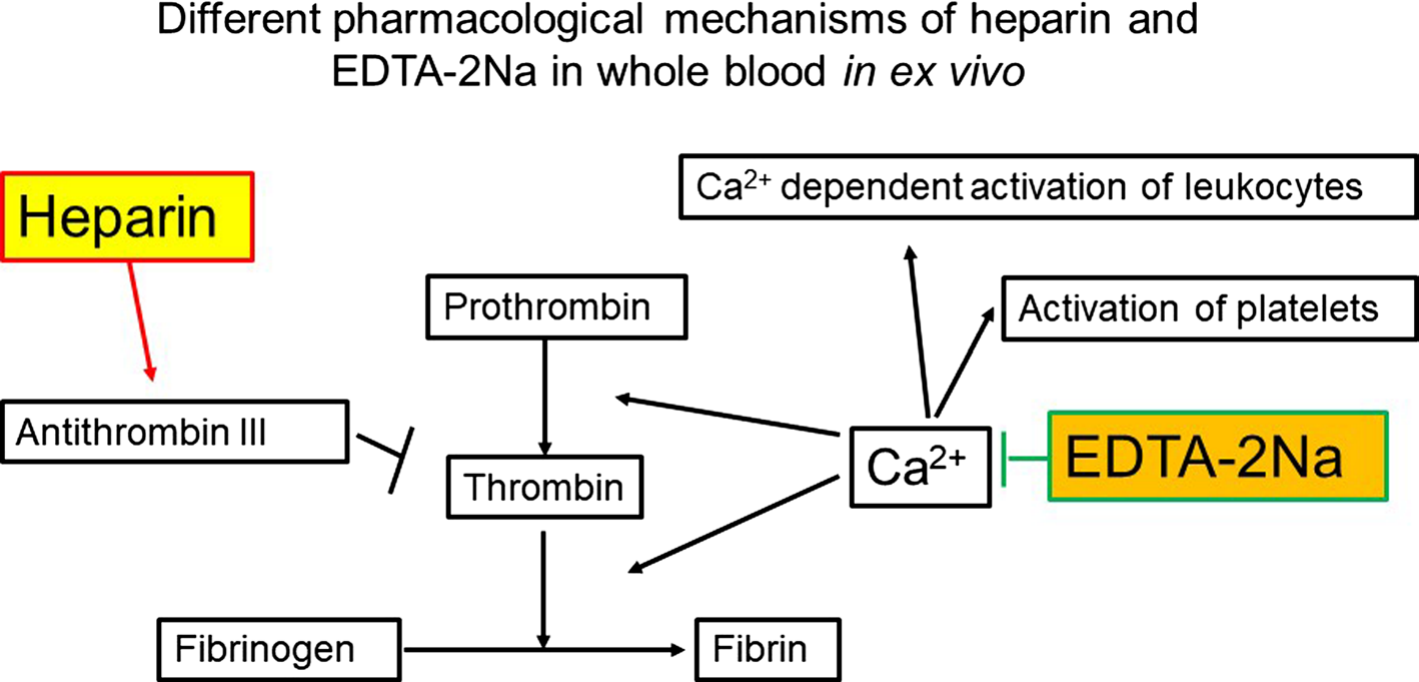
Different pharmacological mechanisms of heparin and ethylene-diamine-tetraacetic acid (EDTA)-2Na in whole blood ex vivo*.* Pharmacological mechanisms of heparin and EDTA-2Na in whole blood ex vivo are demonstrated in this schematic. Heparin binds to the enzyme inhibitor antithrombin (AT) III and greatly accelerates the rate at which AT III inactivates the coagulation enzymes thrombin and factor Xa. In contrast, EDTA-2Na scavenges metal ions (Ca^2+^ and Mg^2+^), thereby inhibiting activation of platelets and Ca^2+^ dependent activation of leukocytes. The heparinized blood tube maintained leukocyte and platelet function ex vivo*,* just as it did in vivo

**Fig. 2:**
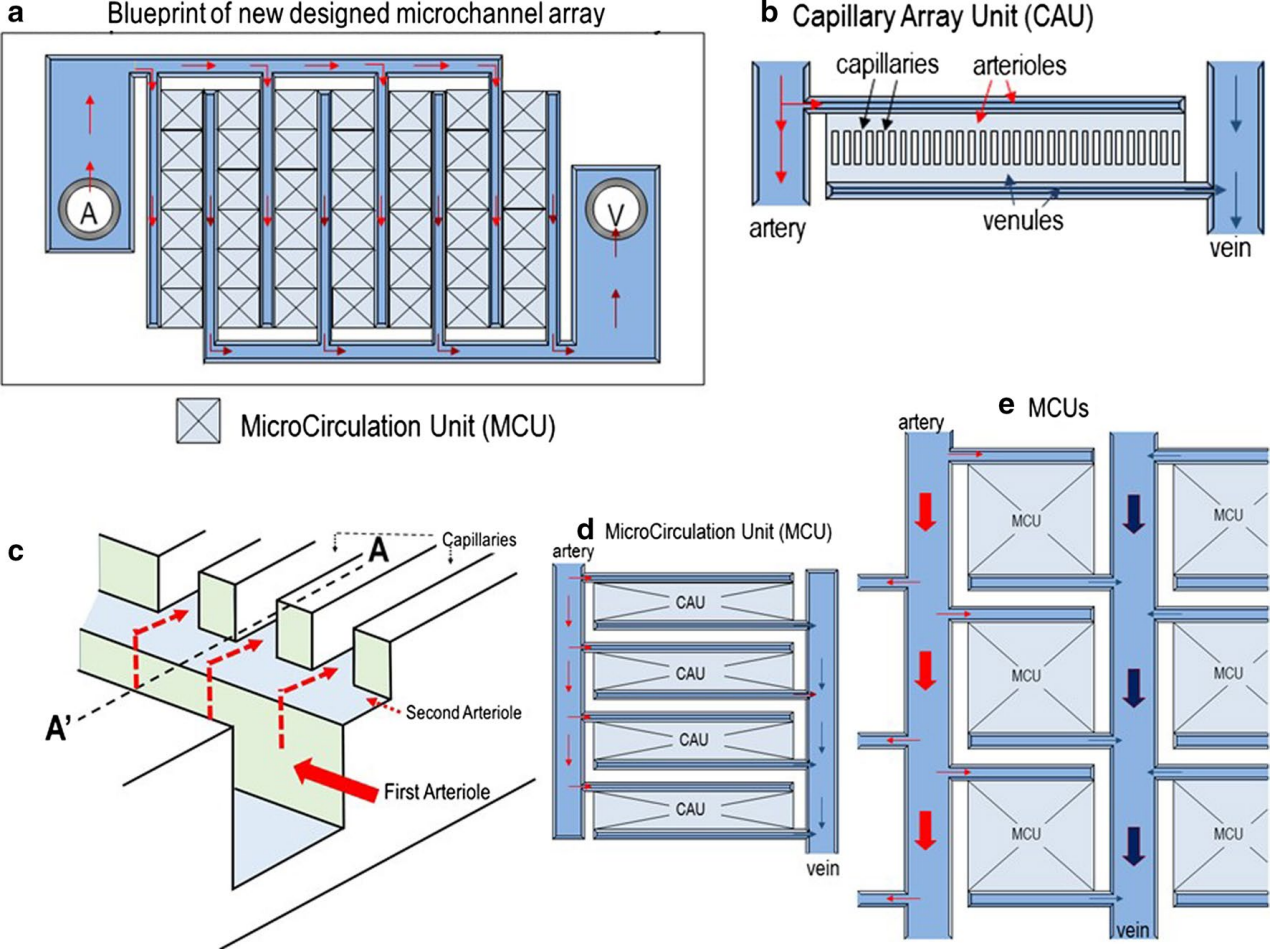
Blueprint of new microchannel array (DKAMCM1-60-7-4.5D) designed to mimic the human microvessel network. **a** Blood sample enters from hole A (artery) into the microchannel array and exits by hole V (vein). **b** A capillary array unit (CAU) comprises 40 capillaries (black arrows) with length, width, and depth of 60, 7, and 4.5 µm, respectively, and each capillary is connected to an arteriole (red arrows) and venule (blue arrows) with length, width, and depth of 640, 30, and 4.5 (light blue) or 50 (blue) µm, respectively. **c** Three-dimensional blueprint of connection of primary arterioles (wide red arrow, 50 µm depth) to secondary arterioles (narrow dashed red lines, 4.5 µm depth), and then to capillaries (narrow red arrows, 4.5-µm depth). The blood sample flows through primary arterioles, secondary arterioles, and capillaries following the red arrows. **d** A microcirculation unit (MCU) comprising four CAUs. **a**, **e** The 42 MCUs are connected via arteries (red arrows) and veins (dark blue arrows) with a depth of 50 µm. Blood sample enters from the artery into MCUs and exits via the vein

**Fig. 3:**
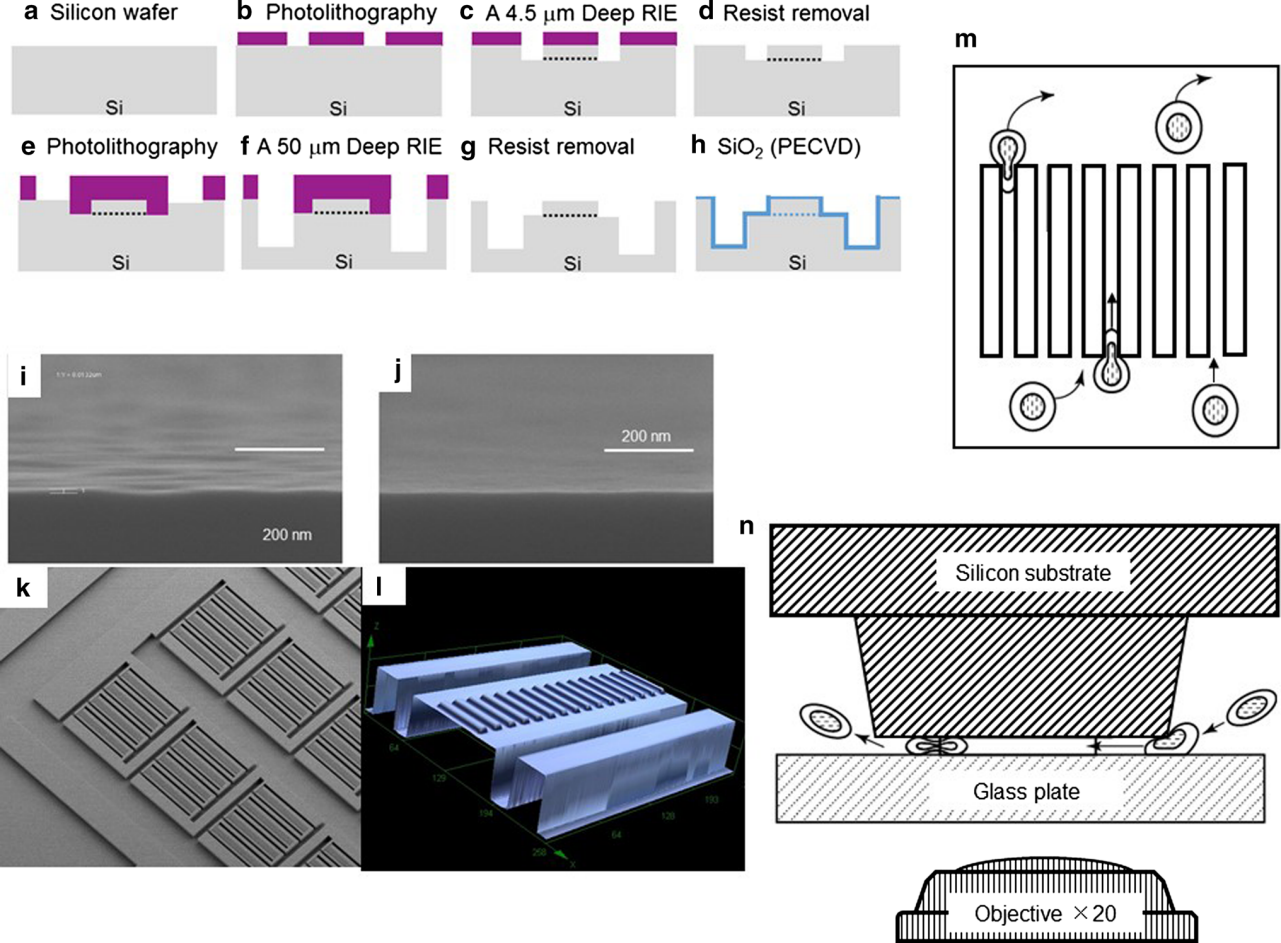
Fabrication process for the microchannel array. **a**, **b**, **e** The designed pattern was machined onto a photomask blank, and an *i*-line stepper was used to transfer the photomask pattern onto photoresist-coated 4-in. silicon wafers. **c**, **f** After the development of the photoresist, inductively coupled plasma dry-etching was used to pattern the silicon wafers with sulphur hexafluoride etchant gas. **g** Diluted potassium hydroxide (KOH) solution was used to slightly etch the patterned silicon to obtain a smoother surface after resist removal. **h** After fabrication, 1-μf-thick silicon dioxide (SiO_2_) was deposited in deep trenches by plasma-enhanced chemical-vapor deposition (PECVD). **i**, **j** The bottom of a 50 μm trench was imaged by scanning electron microscopy (SEM) before **i** and after **j** slight etching by diluted KOH solution. **k**, **l** Microchannel array was carefully examined by SEM (**k**) and by confocal laser microscopy (**l**). **m**, **n** Erythrocytes and leukocytes, which are larger and more viscous than erythrocytes, are transiently deformed at the capillaries. Schematic of leukocytes passing through capillaries from above (**m**) and from the side (**n**) are shown

The pharmacological mechanisms of two commonly used anti-coagulants, heparin and ethylene-diamine-tetraacetic acid (EDTA)-2Na, are demonstrated in whole blood ex vivo (Fig. 1). A blueprint of the new microchannel array (DKAMCM1-60-7-4.5D, National Institute of Advanced Industrial Science and Technology, Tsukuba, Ibaraki, Japan) designed to mimic the human microvessel network employed in this work, is shown in Fig. 2. The designed pattern was machined onto a photomask blank, and an i-line stepper (Model NSR-2205i12D, Nikon, Tokyo, Japan) was used to transfer the photomask pattern onto photoresist, coated onto 4-in. silicon wafers (Fig. 3a, b, e). After the development of the photoresist, an inductively coupled plasma dry-etching system (Model RIE-101iPHS-L, SAMCO, Tokyo, Japan) was used to pattern the silicon wafers with sulphur hexafluoride etchant gas (Fig. 3e, f). To set up the test blood supply and recovery tubes in the silicon chips, holes with a 1.5 mm diameter were machined through the silicon wafers by maskless photolithography (Model DL-1000, Nano System Solutions, Inc., Okinawa, Japan) and the inductively coupled plasma dry-etching system described above. Diluted KOH solution was used to slightly etch the patterned silicon to obtain a smoother surface (Fig. 3i, j). To form a blood-inert surface, the wafers were then introduced into a plasma chemical-vapor deposition system (Model PD-20SS, SAMCO), and a silicon dioxide film with a thickness of 0.4–1.0 µm was deposited at 350 °C by a mixture of tetraethoxysilane and oxygen (Fig. 3h). This film, on the silicon wafers, provided phase contrast that facilitated clear microscopic observation of blood cells, especially transparent leukocytes and platelets. The wafers were then cut into pieces of 8 × 16 mm^2^ each using a dicing saw (Model DAD522, DISCO, Tokyo, Japan) to obtain the microchannel chips. The resulting pattern was carefully examined with a scanning electron microscope **(**Model S-4800, Hitachi High-technologies, Tokyo, Japan) (Fig. 3k) and confocal laser microscope (Model LEXT OLS4100, Olympus Corp., Tokyo, Japan) (Fig. 3l). Schematic of leukocytes passing through capillaries from the above and from the side are shown in Fig. 3m, n, respectively.

**Fig. 4:**
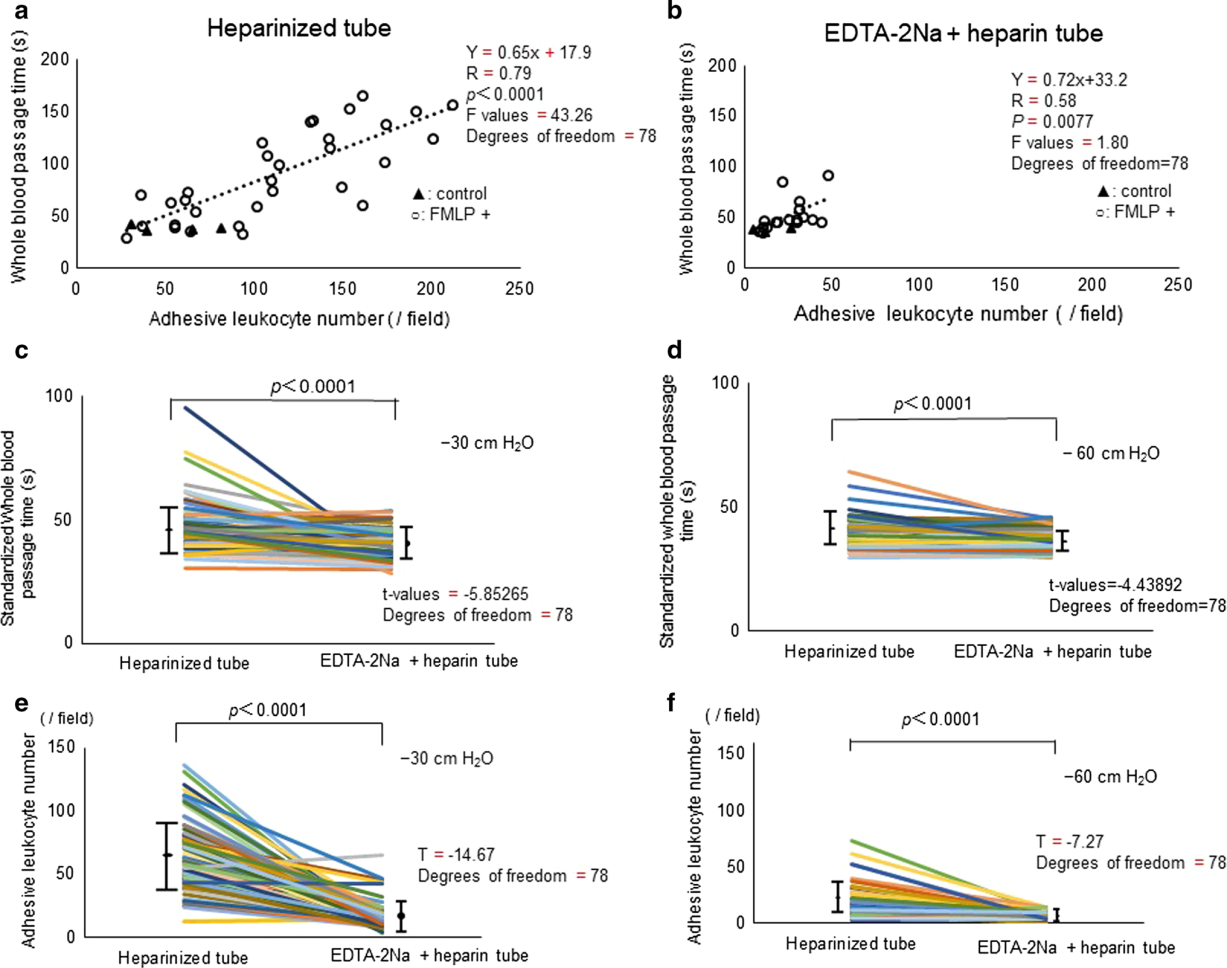
Whole blood passage time through microchannel arrays and adhesive leukocytes. **a**, **b** There was a significant correlation between passage time of heparinized whole blood through microchannel arrays and adhesive leukocyte number per field (**a***r* = 0.79, *p* < 0.0001) and those of ethylene-diamine-tetraacetic acid (EDTA)-2Na + heparinized blood (**b***r* = 0.58 *p* = 0.0077) with (filled triangle)/without (open circle) 10^–9^ or 10^–10^ M formyl-methionyl-leucyl-phenylalanine (FMLP) to activate leukocytes. FMLP significantly increased the numbers of adherent or clumped leukocytes and significantly increased the duration of whole blood passage. **c**, **d** Standardized passage time of whole blood with heparin and blood with heparin + EDTA-2Na under constant vacuum of 30 cmH_2_O and 60 cmH_2_O. Note that the passage time of whole blood with heparin + EDTA-2Na was significantly shorter (*p* < 0.0001) than that of whole blood with only heparin under both suction pressures. **e**, **f** The adhesive leukocyte number (/field) in blood with heparin + EDTA-2Na was remarkably less than that in blood with heparin alone under both suction pressures (*p* < 0.0001)

First, we determined whether our new BWA-MCFAN-equipped method with DKAMCM1-60-7-4.5D could quantify leukocyte activation induced by *N*-formyl-methionyl-leucyl-phenylalanine (FMLP) ex vivo. FMLP was added to fresh whole blood from healthy subjects to activate leukocytes experimentally [19, 20], and then the whole blood passage time was measured by BWA-MCFAN. The addition of FMLP to heparinized blood significantly increased the whole blood passage time and adhesive leukocyte number, which were significantly correlated (Fig. 4a). However, the whole blood passage time and adhesive leukocytes in blood treated with EDTA-2Na and heparin only slightly increased after FMLP application (Fig. 4b).

**Fig. 5:**
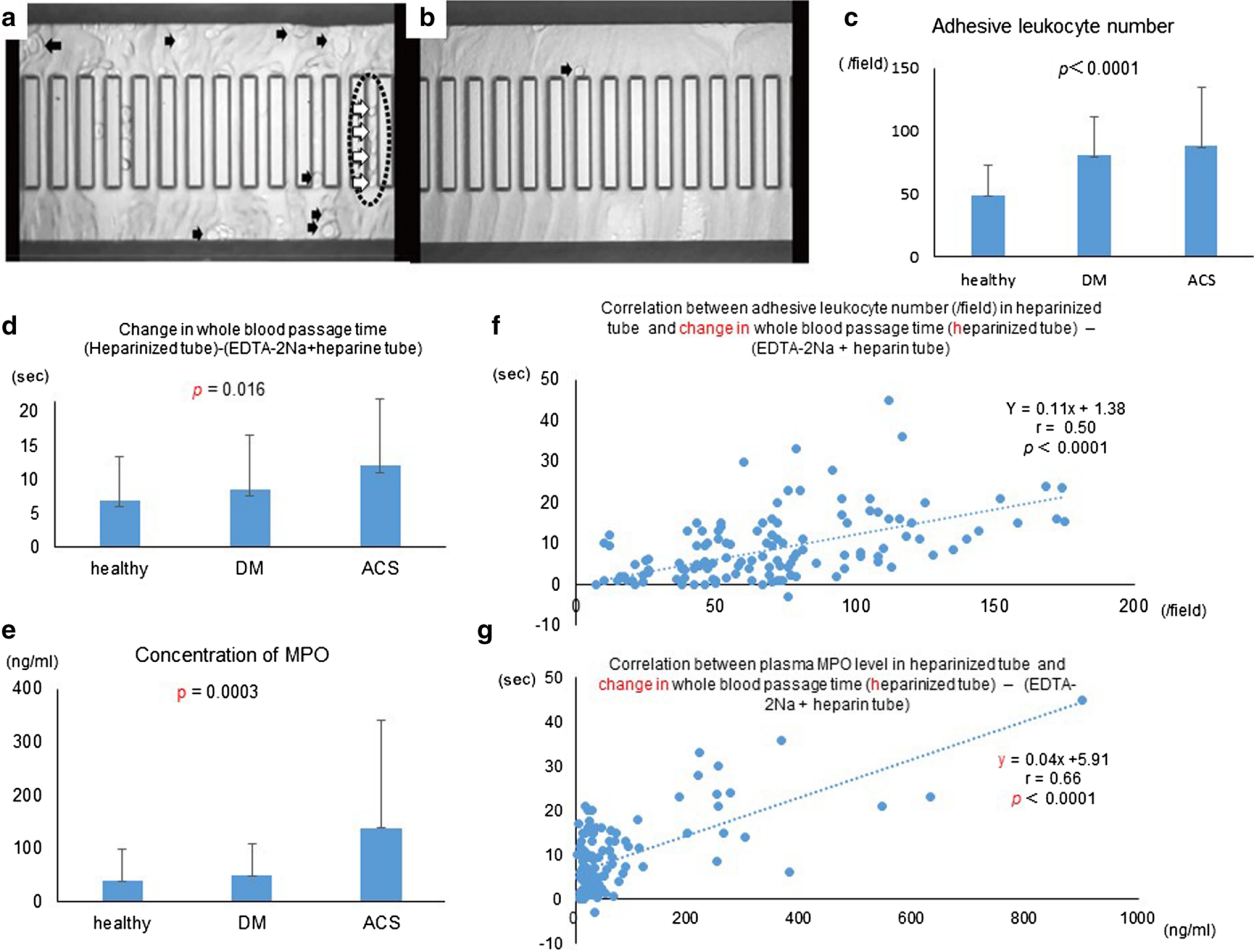
Microphotographs of whole blood passing through microchannel arrays Blood was sampled from the same healthy subject (**a**, **b**). **a** Adherent or clumped leukocytes (black arrows) were observed in whole blood with heparin in the microchannel flow analyser (MCFAN) by high-powered field (× 1400)*.* Deformed erythrocytes running through capillaries (white open arrows) were visible in the high-powered field (**a**). **b** There was no platelet aggregation and significantly fewer adherent leukocytes (black arrow) in blood with ethylene-diamine-tetraacetic acid (EDTA)-2Na + heparin because of Ca^2+^ scavenging by EDTA-2Na. **c** The number of adhesive leukocytes was significantly lower (*p* < 0.0001) in healthy subjects (*n* = 79) than those subjects with diabetes mellitus (DM) (*n* = 42) and acute coronary syndrome (ACS) (*n* = 36). **d** Change in whole blood passage time (heparinized tube) − (EDTA-2Na + heparin tube) in patients with DM and ACS were increased compared samples from healthy subjects. **e** Plasma concentration of myeloperoxidase (MPO) of healthy subjects was lower than those of DM and ACS subjects. **f** There was a significant correlation between change in whole blood passage time {(heparin tube) − (EDTA-2Na + heparin)} and adhesive leukocyte number (heparin) under − 30 cmH_2_O (*r* = 0.50, *p *< 0.0001). **g** There was also a significant correlation between change in whole blood passage time and plasma levels of MPO (*r* = 0.66, *p* < 0.0001)

Next, we confirmed that passage times of whole blood with heparin + EDTA-2Na were always shorter than those of whole blood with only heparin in healthy subjects (*n* = 79, Table 1) under both suction pressures of − 30 cmH_2_O and − 60 cmH_2_O **(**Fig. 4c, d, Videos 1, 2**)**. There were variations in the number of adhesive leukocytes per field in heparinized blood below − 30 cmH_2_O (Fig. 4e) and fewer below − 60 cmH_2_O **(**Fig. 4f**)**. Numbers of adhesive leukocytes in blood with heparin + EDTA-2Na were remarkably lower than those in blood with only heparin under both suction pressures (Fig. 4e, f, Videos 1, 2). Microphotographs (Fig. 5a, b) and movies (Videos 1, 2) clearly showed deformed erythrocytes flowing through the capillary microarrays in a high-powered field, similar to the in vivo condition.

The adhesive leukocyte number per field in blood with heparin was significantly lower (*p* < 0.0001) in healthy subjects (*n* = 79) than in patients with Type 2 diabetes mellitus (DM, *n* = 42) and acute coronary syndrome (ACS, *n* = 36, Fig. 5c). Changes in whole blood passage time {(heparinized tube) – (EDTA-2Na + heparin tube)} in patients with DM and ACS were increased compared to those of healthy subjects (Fig. 5d). Similarly, plasma concentration of MPO of healthy subjects was lower than those of DM and ACS subjects (Fig. 5e). There was a significant correlation between change in whole blood passage time {(heparin tube) − (EDTA-2Na + heparin)} and adhesive leukocyte number (heparin) under − 30 cmH_2_O (*r* = 0.50, *p *< 0.0001) (Fig. 5f). There was also a significant correlation between change in whole blood passage time and plasma levels of MPO (*r* = 0.66, *p *< 0.0001) (Fig. 5g).

**Fig. 6:**
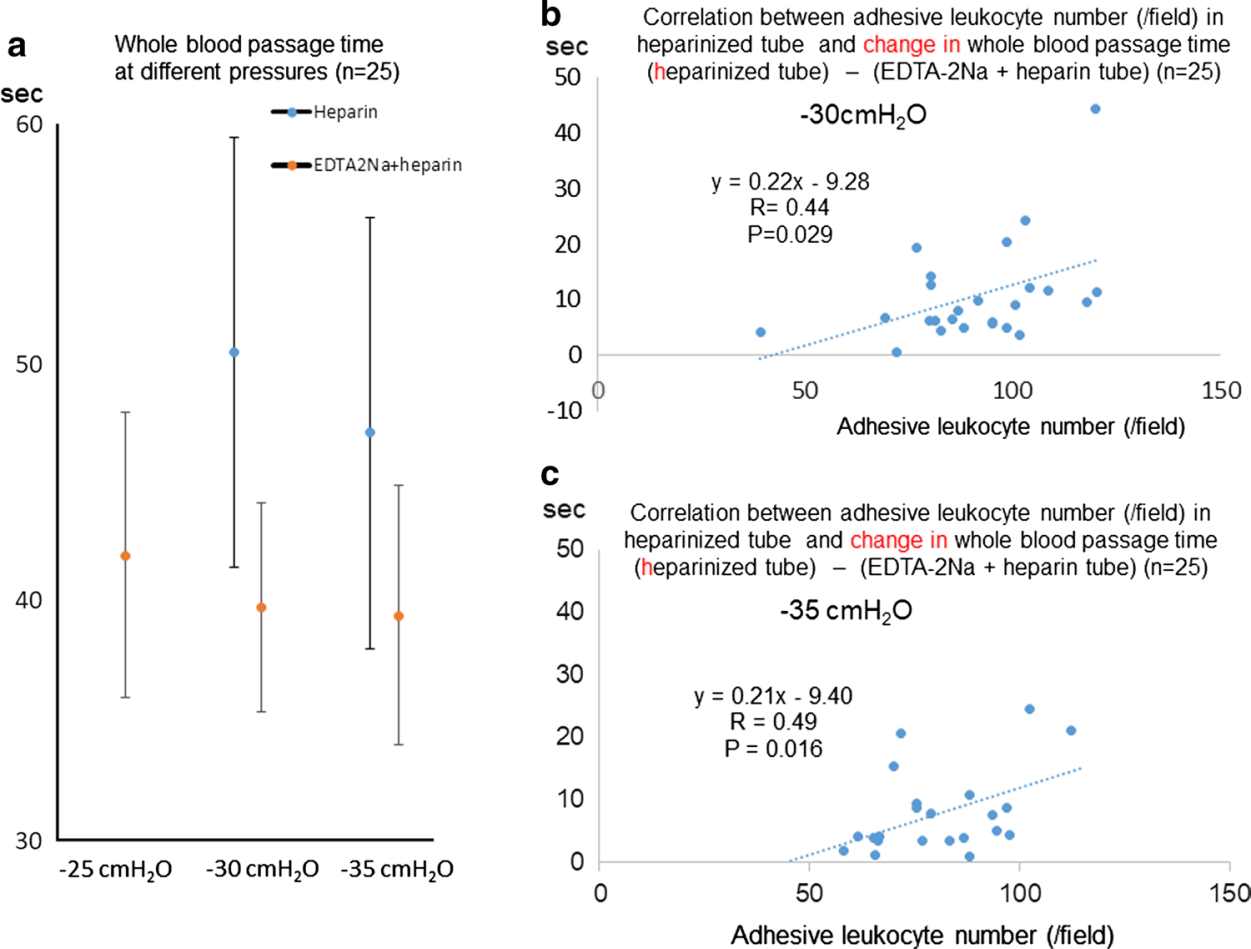
Passage times of whole blood through microchannel arrays under − 25 cmH_2_O, − 30 cmH_2_O, and − 35 cmH_2_O. Standardized passage times of whole blood with heparin and whole blood with heparin + EDTA-2Na under a constant vacuum of 25 cmH_2_O, 30 cmH_2_O, or 35 cmH_2_O in 25 subjects (**a**). The passage time of whole blood with heparin under a constant vacuum of 25 cmH_2_O could not be measured in 19 of 25 subjects because of obstruction of the microchannel arrays. The passage time of whole blood with heparin + EDTA-2Na was shorter than that of whole blood with heparin under both 30 cmH_2_O and 35 cmH_2_O suction pressures. There was a significant correlation between change in whole blood passage time {(heparin) − (EDTA-2Na + heparin)} and adhesive leukocyte number (heparin) under − 30 cmH_2_O (**b***r* = 0.44, *p* = 0.029) and − 35 cmH_2_O (**c***r* = 0.49, *p* = 0.016)

Additionally, we measured the passage times of whole blood with heparin and whole blood with heparin + EDTA-2Na under a constant vacuum of 25 cmH_2_O, 30 cmH_2_O, or 35 cmH_2_O in 25 subjects to determine the best pressure for assessing leukocyte activity (Fig. 6). The passage time of whole blood with heparin under a constant vacuum of 25 cmH_2_O could not be measured in 19 of 25 subjects because of obstruction of many microchannel arrays by activated leukocytes and platelets. The optimal pressure for assessing leukocyte activity may be 30 cmH_2_O and/or 35 cmH_2_O.

## Discussion

We developed a clinically feasible method for assessing leukocyte rheology in whole blood using a MCFAN with a novel silicon chip designed to mimic the human microvascular network. We designed and developed a new multi-manifold microchannel array chip, DKAMCM1-60-7-4.5D, using dry-etching, resulting in a square valley to observe how blood cells pass through the capillary tube when they are deformed. The silicon dioxide film on the silicon wafers provided both a blood-inert surface and phase contrast for microscopic observation of moving blood cells [18], even erythrocytes and leukocytes transiently deformed at the capillaries, agglomerated in the capillaries, or adhered to post-capillary venules (Videos 1, 2).

In addition, the present new microchannel array chip prevents artefactual activation of platelets by a stepwise decrease in microchannel diameter (Video 1). This artefactual activation of platelets downstream of capillaries induced by conventional microarray chips with abrupt narrowing may prolong to the whole blood passage time somewhat [17].

In the presence of Ca^2+^, leukocytes can transform to an active state with pseudopods and increased viscoelastic coefficients [7, 19, 20, 23, 24]. FMLP increases Ca^2+^ levels in human neutrophils in a concentration-dependent manner, as determined by Fura-2 imaging and reactive oxygen intermediates production [20]. In the present study, application of FMLP to heparinized whole blood in the presence of Ca^2+^ significantly increased the adhesive leukocyte number, and EDTA-2Na inhibited FMLP-induced activation of leukocytes and platelets by scavenging Ca^2+^. EDTA inhibits leukocyte activation by (1) inhibition of Ca^2+^-dependent activation of leukocytes, and (2) inhibition of platelet–leukocyte interaction. The inhibition of activation of leukocytes and platelets by EDTA-2Na was associated with an increase in leukocyte deformability and a decrease in leukocyte adhesion to the chip and platelets. The change in the passage time of whole blood, which is significantly correlated with the adhesive leukocyte number and plasma levels of MPO, was calculated using BWA-MCFAN by subtraction of the passage time of whole blood treated with EDTA-2Na + heparin from that of blood treated with heparin only, even in blood from patients with DM and ACS, who suffered from inflammation. This technique may provide a clinically feasible method for determination of leukocyte rheology. The rheology of leukocytes has significant implications for their functional behaviour, including flow through, microcirculation, and interaction with endothelial cells [24–26]. The optimal pressure for assessing leukocyte activity may be 30 cmH_2_O and/or 35 cmH_2_O.

In conclusion, we have developed a clinically feasible method for assessing leukocyte rheology in whole blood using BWA-MCFAN with a new silicon chip designed to mimic the human microvascular network. Using the anticoagulants heparin and EDTA-2Na, we were able to quantify leukocyte rheology and show that the change in whole blood passage time, as calculated by subtraction of the passage time of blood treated with both heparin and EDTA-2Na from that of blood treated with heparin only, is correlated with adhesive leukocyte number.

## Electronic supplementary material

Below is the link to the electronic supplementary material.

Supplementary file1 Microscopic video demonstrating heparinized whole blood, from a 30-year-old healthy male current smoker, passing through the microchannel array under − 30 cmH_2_O. Note that many activated leukocytes adhered on the terrace and clumped in the capillaries. As a result, whole blood passage time was prolonged (57 s). Videos 1 and 2 feature samples from the same subject with a body mass index of 22, haematocrit of 47, and white cell count of 7330/ml (MP4 1387 kb)

Supplementary file2 Microscopic video demonstrating ethylene-diamine-tetraacetic acid (EDTA)-2Na and heparinized whole blood from the same subject as for the sample in Video 1 passing through the microchannel array under − 30 cmH_2_O. Note that fewer leukocytes adhered on the terrace and clumped in the capillaries than in the heparin-only sample featured in Video 1. Whole blood passage time was 43.6 s (MP4 1420 kb)

## Data Availability

The datasets generated during and/or analysed during the current study are available from the corresponding author on reasonable request.
